# Acute Hemorrhage into Adrenal Pseudocyst Presenting with Shock: Diagnostic Dilemmas — Report of Three Cases and Review of Literature

**DOI:** 10.1100/tsw.2006.369

**Published:** 2006-03-19

**Authors:** Amarapathy Sivasankar, Sathyanesan Jeswanth, Maria Antony Johnson, Palaniappan Ravichandran, Shanmugasundaram Rajendran, Devy Gounder Kannan, Rajagopal Surendran

**Affiliations:** Department of Surgical Gastroenterology, Stanley Medical College Hospital, Chennai, Tamilnadu, India

**Keywords:** adrenal glands, pseudocyst, hemorrhage, pregnancy

## Abstract

We report three cases of acutely bleeding adrenal pseudocysts presenting as hemorrhagic shock. Pregnancy was associated in two cases. The diagnostic dilemmas are discussed with special reference to their unusual presentations, diagnosis, and treatment. We believe that our cases, complicated by intracystic hemorrhage, may be related to pregnancy.

## INTRODUCTION

Adrenal pseudocysts are cystic lesions arising within the adrenal gland surrounded by a fibrous tissue wall and devoid of a recognizable lining layer. We report three cases of adrenal pseudocysts that presented with a state of shock secondary to massive hemorrhage into the pseudocysts.

## CASE SUMMARIES

### Case 1

A 20-year-old pregnant lady presented with acute pain in the right hypochondrium and right lumbar region followed by giddiness in the $8^{th}$ week of pregnancy. The patient was pale and in a state of shock. Examination of the abdomen revealed a large tender mass occupying the right hypochondrium and lumbar region. Hemoglobin was 4.2 gm/dl. She received 6 units of blood transfusion. CT scan showed a 11- × 15-cm sized, heterodense, well-defined lesion with predominant cystic component extending from the lower border of the liver up to the right iliac crest with multiple septations displacing the right kidney posteriorly (Fig. 1). The fat plane between the mass and liver could not be identified. A single viable fetus

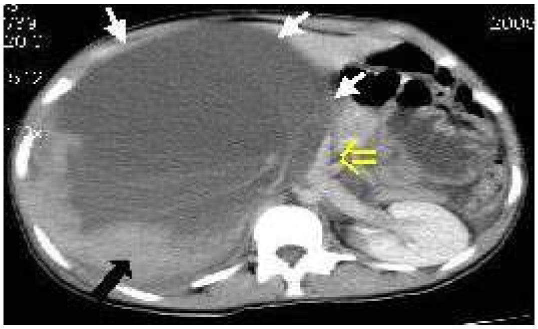

**FIGURE 1.** Contrast-enhanced CT revealing a very large right retroperitoneal mass with higher density representing large hematoma with fresh hemorrhage (black arrow) and organizing hematoma (white arrows). Mass displacing the right kidney and IVC to the left side (open arrow).

of 8 weeks gestation was seen. The differential diagnosis included a bleed into a hepatic adenoma or retroperitoneal cyst. Emergency laparotomy revealed a large retroperitoneal hemorrhagic cyst measuring 20 cm in diameter closely apposed to the right lobe of the liver and contained 5 l of hemorrhagic fluid with blood clots. Complete cyst excision was done. Her postoperative course was uneventful. Pregnancy was terminated due to fear of fetal teratogenicity because of exposure to radiation and drugs used for resuscitation. Microscopic examination of the cyst wall demonstrated strips of loose fibrovascular tissue with fibroblastic proliferation with foci of smooth muscle and adipose tissue and areas of hemorrhage (Fig. 2) consistent with hemorrhagic adrenal pseudocyst.

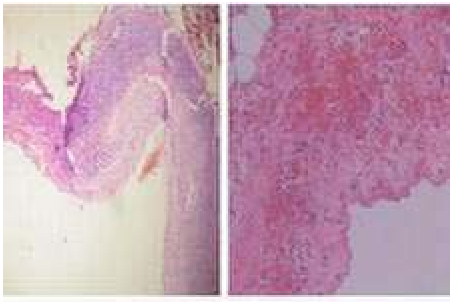

**FIGURE 2**. (Left) A representative cross-section of the entire cyst within the adrenal gland. (H-E stain); (right) many strips of loose fibrovascular tissue with some fibroblastic proliferation with extensive hemorrhage.

## Case 2

A 24-year-old pregnant lady presented in a state of shock following an acute abdominal pain in the 20$^{th}$ week of pregnancy. She was pale, and abdominal examination revealed fullness and tenderness in the right hypochondrium. Hemoglobin was 7.2 gm/dl. She received 6 units of blood. CT scan demonstrated a cystic mass of 14- × 16-cm size that appeared to arise from the lower portion of the liver with hyperdense areas within the mass. MRI showed a large cystic mass in the right-side upper abdomen extending into the right lumbar region and right iliac fossa with areas of hypointensity on T2-weighted sequences suggesting intralesional hemorrhage (Fig. 3). Preoperative diagnosis of bleeding liver tumor was made. At exploratory laparotomy, an adrenal cyst 15 cm in diameter was seen (Fig. 4). Three liters of hemorrhagic fluid was drained. Complete cyst excision was done. The postoperative course was uneventful. Pregnancy was terminated because of exposure to radiation and drugs used for resuscitation. Histology confirmed the lesion as hemorrhagic adrenal pseudocyst.

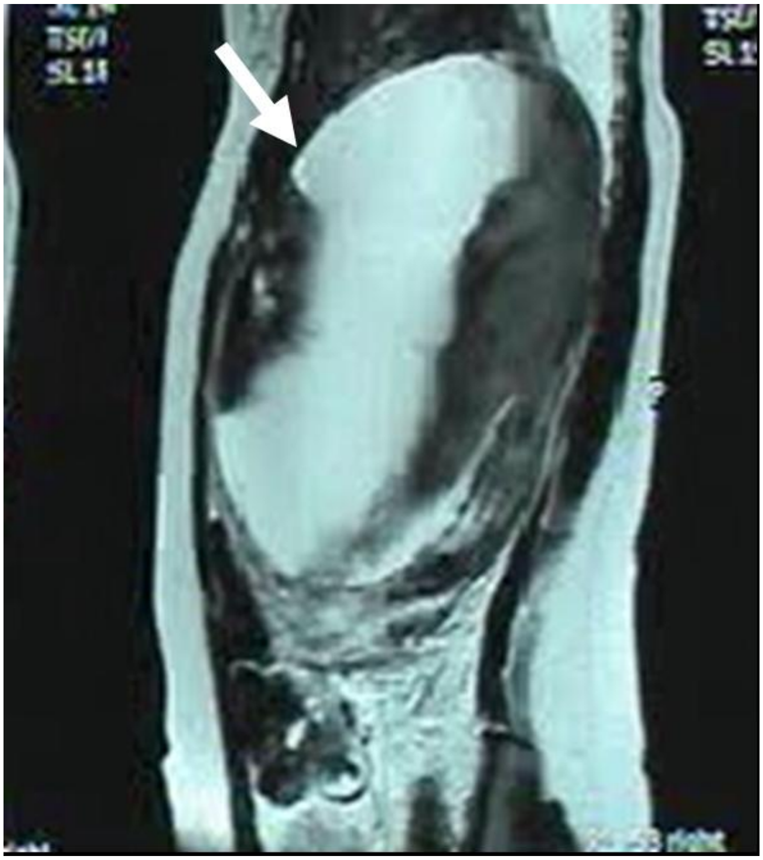

**FIGURE 3.** MRI shows a large mass (white arrow) in right-side upper abdomen extending into right lumbar and right iliac fossa with areas of hypointensity, measuring 14 × 16 cm in size.

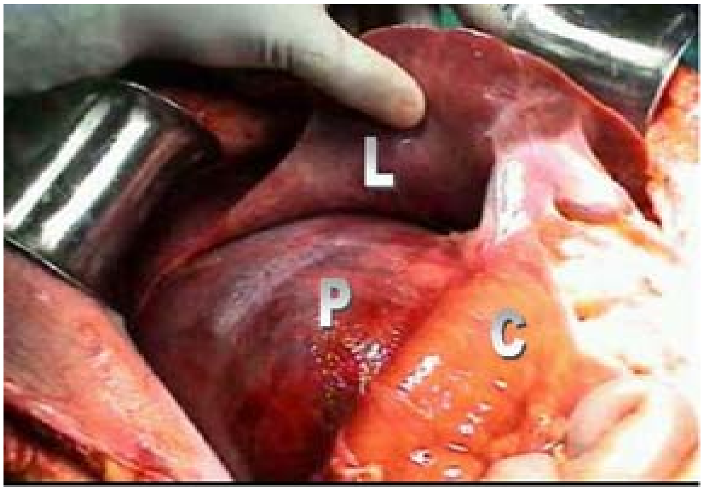

**FIGURE 4.** Operative photograph shows large hemorrhagic retroperitoneal cyst arising from right adrenal gland. (L = liver, C = colon, P = pseudocyst).

## Case 3

A 26-year-old lady diagnosed with an infected hydatid cyst of liver elsewhere, presented with acute, right-upper abdominal pain and in a state of shock. She was pale and there was tenderness in the right hypochondrium. Hemoglobin was 6 gm/dl. She received 8 units of blood. CT scan demonstrated a cystic mass of 16- × 18-cm size in the right lobe of the liver with calcification of wall and areas of hemorrhage. Based on clinical and imaging findings, bleeding into a cyst of the liver was suspected. Transperitoneal laparoscopy revealed an adrenal cyst of 16-cm diameter. Three liters of hemorrhagic fluid was drained. Marsupialization of the cyst was done. The postoperative course was uneventful. Histologically, the lesion was proved to be a hemorrhagic adrenal pseudocyst. Table 1 summarizes the demographic and clinical data of the three patients.

**TABLE 1**
**Patient Demographics and Clinical Data**

| | Case 1 | Case 2 | Case 3 |
|---|---|---|---|
| Sex | Female | Female | Female |
| Age (years) | 20 | 24 | 26 |
| Presenting symptoms | Right upper quadrant pain, shock | Right upper quadrant pain, shock | Right upper quadrant pain, shock |
| Pregnancy | + | + | – |
| Preoperative cyst diagnosis (based on clinical and imaging findings) | Bleeding hepatic adenoma/focal nodular hyperplasia/retro-peritoneal cyst | Bleeding hepatic adenoma/focal nodular hyperplasia | Hemorrhagic liver cyst |
| Side | Right | Right | Right |
| Cyst size (cm) | 20 | 15 | 12 |
| Operative time (min) | 120 | 135 | 150 |
| Procedure | Open cyst excision | Open cyst excision | Laparoscopic marsupialization |
| Hospital stay (days) | 8 | 9 | 2 |
| Pathological findings | Hemorrhagic adrenal pseudocyst | Hemorrhagic adrenal pseudocyst | Hemorrhagic adrenal pseudocyst |
| Follow-up (months) | 6 | 14 | 18 |

## DISCUSSION

Adrenal cysts are rare[1] (0.064–0.18% in autopsy series), and fewer than 500 cases have been reported in the western literature. Most adrenal cysts are small. Cysts are found two to three times more frequently in women than in men and bilateral in approximately 10% of cases. The 5$^{th}$ and 6$^{th}$ decades of life are the usual ages at clinical presentation. All the three cases in the present series were women in the 3$^{rd}$ decade. Endothelial-lined cysts and pseudocysts are the most common types of adrenal cyst and comprise approximately 90% of cases. All the three cases in the present series had pseudocysts.

A pseudocyst is defined as a non-neoplastic, nonparasitic cyst of the adrenal gland in which an epithelial or endothelial lining is not demonstrated. Pseudocysts are generally unilocular and the liquid present inside them has a reddish color. They can reach large sizes similar to the cases in the present series. Pseudocysts represent the common variety of adrenal cysts that are discovered clinically. Pseudocysts may be found incidentally on a routine imaging workup.

Adrenal pseudocysts can rarely present along with a state of shock secondary to a massive hemorrhage similar to the three cases in the present series[2]. The bleeding may follow a blunt trauma to the lumbar region, leading to voluminous hematomas in retroperitoneum[3]. Frequently, calcification is present in the wall of the cyst. In a series of 33 calcified adrenal masses, 18% were cysts, 6 of the 8 adrenal cysts had mural calcification[4]. Calcification is particularly found in echinococcus cysts and pseudocysts resulting from hemorrhage and necrosis in adrenal tumors. One patient in the present series had calcification of the pseudocyst.

Adrenal cysts have been reported to be associated with hepatic focal nodular hyperplasia[5], hypertension, and pregnancy[6,7,8,9]. Pseudocysts are rarely encountered during pregnancy, but it should be considered as an important differential diagnosis of a mass presenting with an acute, right-side upper abdominal and flank pain[6,7,8,9] and/or with a hemorrhagic shock during pregnancy. Pregnancy and trauma of parturition may result in hemorrhagic adrenal pseudocyst[8]. We believe that our cases, complicated by intracystic hemorrhage, may be related to pregnancy. The outcome of pregnancy appears unaffected by such cysts[9]. Two of our patients had termination of pregnancy due to fear of fetal teratogenicity because of exposure to radiation and drugs used for resuscitation.

Adrenal cysts can pose diagnostic problems. Clinical and imaging findings may be deceptive[10,11] as in two of our cases. Fine-needle aspiration may not distinguish a benign from a malignant lesion. Surgical biopsy is essential for confirmation of diagnosis. In a series of adrenal cystic lesions, pseudocysts have been associated with an adrenal neoplasm in 19%, of which two cases were adrenal cortical carcinomas[12].

Imaging modality such as ultrasound, CT, and MRI[1] have diagnostic sensitivities of 66.7, 80, and 100%, respectively.

CT characteristics of an adrenal pseudocyst include a well-demarcated cyst that does not enhance with intravenous contrast; density of fluid in the cyst measures at water density or much higher depending on the amount of hemorrhagic debris and protein in the fluid. Cystic neoplasm, such as adrenal cortical carcinoma, is characterized by an increased wall thickness and enhancing septations and/or nodules. Focal absence of periglandular fat between the adrenal gland and the large vessels or liver, as well as deviation or compression of the large vessels by the adrenal cyst, may indicate malignancy.

MRI features of adrenal pseudocysts[13] include: (1) a large component with low signal intensity on both T1- and T2-weighted images and (2) a second, smaller, fluid component. Uncomplicated adrenal cysts are low in signal intensity on T1-weighted images and high on T2-weighted images. Complicated or hemorrhagic adrenal cysts may demonstrate variable signal intensity on both T1- and T2-weighted images and may be difficult to differentiate from solid neoplasms. Enhancing nodules or excrescences should raise the possibility of a cystic neoplasm. However, a limitation of MRI is poor sensitivity for detection of calcification.

Adrenal hormone assay of the fluid can provide a clue to the origin of the cyst. It has been demonstrated that a careful hormonal (for functional cyst) and morphofunctional evaluation is indicated in all adrenal cysts, because even when the available diagnostic modalities are combined, they cannot always define the nature of these cysts. No adrenal cyst without an associated adrenal neoplasm has been found to be functioning[12].

Special precautions should be taken to rule out pheochromocytoma since adrenal cysts have been reported with pheochromocytomas[1]. To avoid possible serious complications as a result of cyst aspiration and excision of a pheochromocytoma without adequate preparation, hormonal evaluation before such interventions appear appropriate. In our cases, all of them presented with shock with no apparent clinical findings of pheochromocytoma and were subjected for emergency surgical exploration without laboratory evaluation. However, we recommend that functional assessment should be carried out to rule out pheochromocytoma and other functioning tumors of adrenal gland whenever there is clinical suspicion.

The pathogenesis of the pseudocyst has been postulated to result from prior hemorrhage and subsequent clot organization within a normal adrenal gland[14]. The differential diagnosis of giant hemorrhagic adrenal pseudocysts include cystic lesions in the adjacent organs such as the liver, spleen, and kidneys, cystic retroperitoneal tumors.

The management of adrenal cysts depends on the presentation of clinical and imaging findings. Surgical excision is indicated in a symptomatic, functioning cyst and is complicated either due to bleeding or rupture and suspicion of malignancy and/or large-sized (>5 cm) lesion. Other cysts with benign characteristics may be treated conservatively with regular follow-up by sonography or CT and hormonal evaluation[15].

Complete excision of the adrenal pseudocyst with preservation of remnant adrenal tissue is recommended. Emergency surgical exploration is indicated in complications such as hemorrhage or rupture. Most adrenal pseudocysts can be excised easily with excellent prognosis for the patient. Laparoscopic management of adrenal cysts has been reported in few studies[16,17], which is an attractive option for management of this complex situation, but still to be evaluated. One case in the present series was managed by transperitoneal laparoscopy.

In summary, three cases of acutely bleeding adrenal pseudocysts presenting as hemorrhagic shock is reported. Pregnancy has been causally associated in two cases. It is an important differential diagnosis for an acute, upper abdominal and flank pain; hemorrhagic shock with a cystic mass lesion in the region of adrenal gland on imaging. The combination of clinical, laboratory, and imaging findings is essential. Awareness of this clinical entity among physicians, surgeons, and radiologists is essential for optimum patient care. Prompt surgical intervention, particularly in the setting of massive hemorrhage into the cyst, will save the patient's life.

## BIOSKETCH

**Amarapathy Sivasankar** is currently a senior resident in the Department of Surgical Gastroenterology, Government Stanley Medical College Hospital, Chennai, Tamilnadu. (The department is a high-volume center with a main focus on hepatopancreatobiliary and esophageal surgery.) The author's current interest is on a chronic pancreatitis research project and future interest will be on hepatic transplantation research.
